# Differences in the Number of Intrinsically Disordered Regions between Yeast Duplicated Proteins, and Their Relationship with Functional Divergence

**DOI:** 10.1371/journal.pone.0024989

**Published:** 2011-09-15

**Authors:** Floriane Montanari, Denis C. Shields, Nora Khaldi

**Affiliations:** UCD Conway Institute of Biomolecular and Biomedical Research, School of Medicine and Medical Sciences, and UCD Complex and Adaptive Systems Laboratory, University College Dublin, Dublin, Republic of Ireland; Tulane University Health Sciences Center, United States of America

## Abstract

**Background:**

Intrinsically disordered regions are enriched in short interaction motifs that play a critical role in many protein-protein interactions. Since new short interaction motifs may easily evolve, they have the potential to rapidly change protein interactions and cellular signaling. In this work we examined the dynamics of gain and loss of intrinsically disordered regions in duplicated proteins to inspect if changes after genome duplication can create functional divergence. For this purpose we used *Saccharomyces cerevisiae* and the outgroup species *Lachancea kluyveri*.

**Principal Findings:**

We find that genes duplicated as part of a genome duplication (ohnologs) are significantly more intrinsically disordered than singletons (p<2.2^e^-16, Wilcoxon), reflecting a preference for retaining intrinsically disordered proteins in duplicate. In addition, there have been marked changes in the extent of intrinsic disorder following duplication. A large number of duplicated genes have more intrinsic disorder than their *L. kluyveri* ortholog (29% for duplicates versus 25% for singletons) and an even greater number have less intrinsic disorder than the *L. kluyveri* ortholog (37% for duplicates versus 25% for singletons). Finally, we show that the number of physical interactions is significantly greater in the more intrinsically disordered ohnolog of a pair (p = 0.003, Wilcoxon).

**Conclusion:**

This work shows that intrinsic disorder gain and loss in a protein is a mechanism by which a genome can also diverge and innovate. The higher number of interactors for proteins that have gained intrinsic disorder compared with their duplicates may reflect the acquisition of new interaction partners or new functional roles.

## Introduction

Intrinsically disordered proteins are biologically active proteins containing sequences without stable secondary and/or tertiary structure [1], [2], [3]. Intrinsically disordered sequences have the potential to associate with many partners thanks to multiple possible metastable conformations [4], [5], [6], [7]. There is greater intrinsic disorder in proteins among eukaryote species by comparison with prokaryotes [8], [9]. Intrinsically disordered proteins have been associated with viral virulence [10], with genetic diseases [11] such as skeletal, bone, and neurodegenerative diseases, connective tissue disorders and cancer [12], [13]. Intrinsically disordered regions typically evolve rapidly compared to ordered regions [14]. However, this is not true for all intrinsically disordered regions, such as the regions containing DNA binding sites [14]. Currently very little is known about the origin and the expansion of protein intrinsic disorder. There are several possible mechanisms explaining how genes encoding intrinsically disordered proteins have arisen. These include *de novo* generation [15], [16], lateral and horizontal gene transfer, and gene duplication [17]. Finally repeat expansion is an important mechanism for the evolutionary enlargement of intrinsically disordered regions [18].

There are different ways to predict intrinsically disordered sequences, based on the amino acid sequence features, and the nearby environment of each amino acid [19], [20]. Intrinsically disordered sequences are enriched in the amino acids Glu, Asp, Ser, Lys, Pro and depleted for Tyr, Trp, Phe, Cys, Ile, Leu, Val and His [21], [22], [23]. Intrinsic disorder prediction methods rely on this amino acid composition, but also on the local amino acid environment along the sequence which avoid intra-chain interactions. Some examples include IUPred [24], and DISOPRED2 [19] (see [25] for an overview). IUPRED is a free command-line software, whose efficacy in identifying intrinsic disorder sequences has been demonstrated in numerous studies [26], [27], [28].

It is known that the yeast *S. cerevisiae* has undergone whole genome duplication (WGD) but only a minority of genes have been maintained in a duplicated form [29]. When both copies of the gene are retained, they are referred to as ‘ohnologs’. Ohnologs can undergo independent evolution, allowing neo- or sub-functionalization [30], [31], [32], [33], [34], [35]. Neo-functionalization corresponds to the creation of a new function in one of the ohnologs that did not exist in the ancestor, while sub-functionalization corresponds to the partitioning of the ancestral functions between the ohnologs.

Alternatively, after WGD, one copy can be lost because of functional redundancy, so that only one copy remains, which is referred to as a ‘singleton’. It has been found that some types of protein are more likely to be retained in duplicates after WGD, for example, kinases [36]. The selection favoring the retention of some proteins may also relate in part to dosage sensitivity [37].

An observed trend in the evolution of ohnologs is their observed rapid sequence evolution compared to singletons [38], [39], [40]. This rapid evolution creates the opportunity for the evolution of new gene functions [41].

The consequences for a protein's interactions after gene duplication is of great interest in understanding questions such as how and why new interactions are gained and others lost. Gene duplication may create the freedom for new evolutionary opportunities, since at least one copy may be freer to experiment with new interactors. Indeed it has been shown that one duplicate usually shows significantly more molecular or genetic interactions than the other [42]. It is therefore not surprising that many studies investigated how patterns of protein interaction may vary after duplication [43], [44], [45], [46], [47]. It is estimated for example that as many as half of all interactions may be replaced by new interactions every 300 Myr in yeast [44].

In this work we examined the dynamics of gain and loss of intrinsically disordered regions in ohnolog and singleton proteins to inspect if changes after genome duplication can create functional divergence. Using *Saccharomyces cerevisiae* as a model species of post-WGD, and taking *Lachancea kluyveri* (also known as *Saccharomyces kluyveri*) as a pre-WGD outgroup, we set out to identify the dynamics of creation, elimination and repartitioning of intrinsically disordered sequences between ohnologs after WGD, in comparison to their *L. kluyveri* orthologs. While the precise evolutionary timing of the divergence of these two species is not known, levels of protein divergence between them are higher than those seen between human and fish [48].

We also investigated the impact of such changes in the distribution of sequence disorder with observed differences in patterns of physical protein-protein interactions between ohnologs.

## Results

We carried out the analysis of gain and loss of intrinsically disordered regions on all ohnologs and singletons that possess at least one intrinsically disordered region (see Methods). These included 793 (72%) ohnologs, and 2837 (51%) singletons (Table 1). All interpretations of the gain and loss of intrinsically disordered regions in *S. cerevisiae* are based on a comparison with the pre-WGD outgroup *L. kluyveri*.

**Table 1:** Intrinsically disordered proteins and intrinsically disordered regions in *S. cerevisiae*, comparison between ohnologs and singletons.

	ohnologs	Singletons
Number of proteins having at least 1 intrinsically disordered region	793	2837
Percentage	72%	51%
Average number of intrinsically disordered regions	2.46	1.47
Median	2	1

### Whole Genome duplicated gene pairs are associated with increases in both gain and loss of intrinsically disordered regions

#### More intrinsically disordered regions in *S. cerevisiae* ohnologs compared to singletons

**Figure 1:**
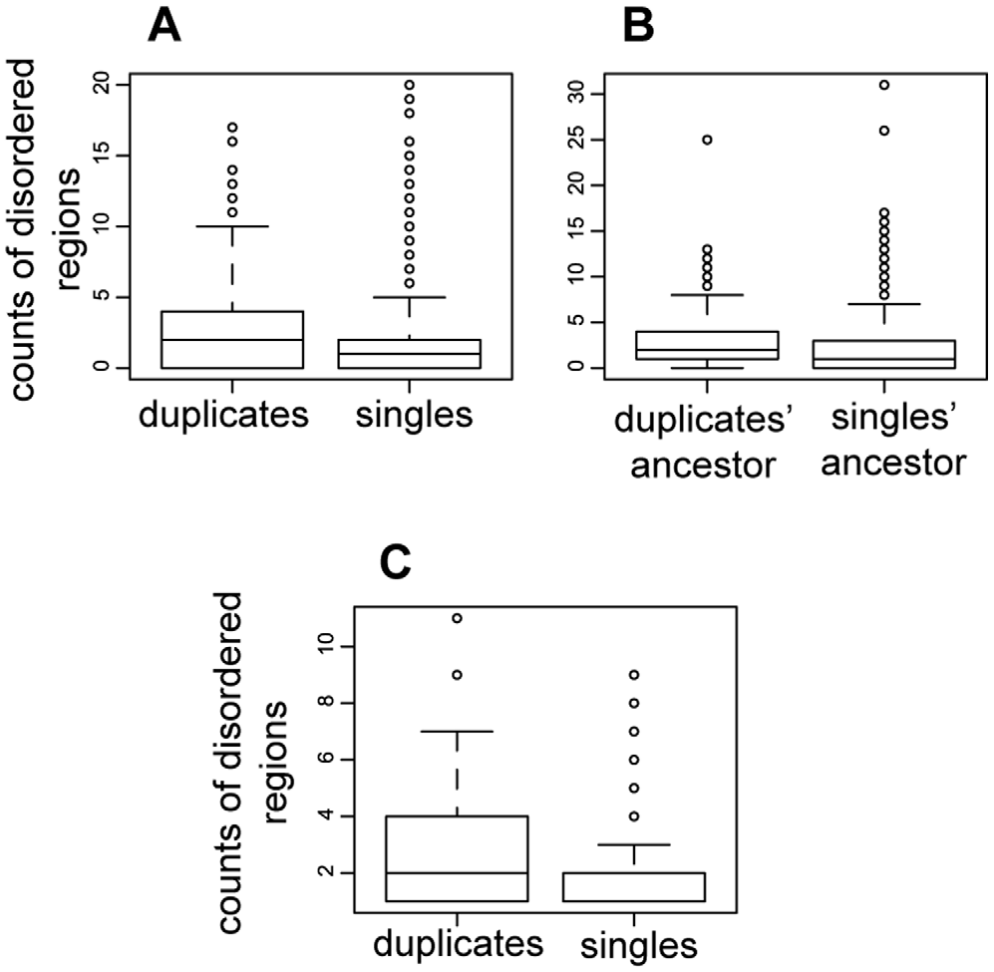
Boxplots of intrinsic disorder acquisition after WGD. (**A**) Boxplot of the absolute counts of intrinsically disordered regions in ohnologs and singletons in *S. cerevisiae*. (**B**) Boxplot of the absolute counts of intrinsically disordered regions in the orthologs of the ohnologs and those of the singletons in *L. kluyveri*. (**C**) Boxplot of the absolute counts of intrinsically disordered regions in the ohnologs and singletons in *S. cerevisiae* after withdrawing the number of regions found in *L. kluyveri* represented in (**B**).

We set out to investigate if the proteins that were retained in duplicate after WGD (ohnologs) have a higher or lower number of intrinsically disordered regions than singleton proteins. We used the number of predicted intrinsically disordered regions as an indicator of their gain and loss in a protein. The results shown in figure 1a and Table 1 indicate that ohnologs have a significantly higher number of intrinsically disordered sequences compared to singletons (p-value<2.2^e^-16). This is not simply a consequence of length differences of the two groups of proteins, since ohnologs are not significantly different in length from singletons (p = 0.09; t-test).

This result is also seen when we alternatively define proteins as intrinsically disordered when they contain one or more intrinsically disordered region: 793 of the total 1100 ohnologs (550 pairs) contain at least one intrinsically disordered region (72%); while for singletons only 2837 of the total 5497 do so (51%). A proportional test supports the same conclusion that there are more intrinsically disordered proteins among ohnologs than among singletons (p<2.2e-16). This is not only statistically significant, but is clearly likely to have some biological relevance, since the percentages are very different.

#### More intrinsic disorder in *L. kluyveri* proteins that were preferentially retained in duplicate form in *S. cerevisiae*

We wanted to understand if this difference reflects changes in intrinsic disorder since duplication, or whether there is a bias in retention of more intrinsically disordered proteins after duplication. This is possible, as it has been shown that certain types of proteins have been favorably maintained in duplicates after WGD [36], [49], [50]. We also note that certain types of proteins in yeast such as regulatory, transcriptional, and developmental proteins tend to be more intrinsically disordered than other types of proteins [6]. When comparing the number of intrinsically disordered regions of the singleton's orthologs in *L. kluyveri* to the orthologs of ohnologs, we find that they are significantly different (p-value = 1.1e-14) with the ortholog of ohnologs in *L. kluyveri* containing more intrinsically disordered regions (figure 1b; Table 2c, p-value = 5.6e-15). This indicates that the excess of intrinsic disorder among duplicates versus singletons may reflect a preference for retaining intrinsically disordered proteins in duplicate.

**Table 2:** Intrinsically disordered proteins and intrinsically disordered regions in *L. kluyveri*, comparison between the set of the duplicates orthologs and the singletons orthologs.

	ortholog of the ohnologs in *L. kluyveri*	ortholog of the singletons in *L. kluyveri*
Number of proteins having at least 1 intrinsically disordered region	407	2517
Percentage	76.5%	62.6%
Average number of intrinsically disordered regions	2.79	1.86
Median	2	1

#### Is the higher intrinsic disorder of ohnologs only due to a retention bias?

The difference seen between ohnologs and singletons therefore is clearly influenced by a bias towards retaining more intrinsically disordered proteins in duplicate. While this is a very interesting observation, we were interested whether this was the only reason behind the current enrichment in intrinsically disordered regions of the ohnologs compared to singletons. To examine this, we subtracted the number of intrinsically disordered regions found in *L. kluyveri* from that of the orthologous proteins in *S. cerevisiae*. This removes, or at least reduces, the bias caused by a preferential retention of intrinsically disordered regions in the ancestor of ohnologs (this is approximated by using the pre-WGD outgroup *L. kluyveri*). We find that ohnologs in *S. cerevisiae* when compared to their ortholog in *L. kluyveri* have significantly more gain than the singletons (p = 0.002, Wilcoxon), with the gain of an average of 0.72 intrinsically disordered region per protein in the ohnologs, as opposed to an average of 0.41 for the singletons (Figure 1-c; Table 3). We found that 29% (154) of ohnologs in *S. cerevisiae* have gained at least one intrinsically disordered region compared to their orthologs in *L. kluyveri*, while the figure is only 25% (1027) for singletons (Table 4). Although the higher number of intrinsically disordered regions in proteins that were preferentially retained in duplicate after WGD is one of the reasons for the current observed intrinsically disordered region enrichment, it does not account for all the enrichment, and WGD seems to have allowed for more intrinsic disorder acquisition in *S. cerevisiae* compared to *L. kluyveri*.

**Table 3:** Average gain and loss of *S cerevisiae* intrinsically disordered sequences (in comparison to *L. kluyveri*) in ohnologs and in singletons.

	Ohnologs	Singletons
Average number of intrinsically disordered regions gained	+0.72	+0.41
Average number of intrinsically disordered regions lost	−1.22	−0.41

The absolute value of the loss in duplicates is significantly higher (p-value = 5.22^e^-4) than the gain.

**Table 4:** Comparison of the gain and loss in intrinsically disordered regions in the ohnologs and the singletons compared with their ortholog in *L. kluyveri*.

	Ohnologs	Singletons
Proteins with identical numbers of intrinsically disordered sequences compared to their ortholog in *L. kluyveri*	33.8% (179)	49.3% (1981)
Proteins with more intrinsic disorder than their ortholog in *L. kluyveri*	29.1% (154)	25.6% (1027)
Proteins with less intrinsic disorder than their ortholog in *L. kluyveri*	37.2% (197)	25.1% (1011)

The higher gain of intrinsically disordered regions in ohnologs compared to singletons, is accompanied with an even greater loss of intrinsically disordered regions (Table 3). In other words, the rate of accumulation or loss of intrinsically disordered regions (compared to *L. kluyveri* orthologs) affects to a greater extent ohnologs compared to singletons (−0.5 ( = −1.22+0.72) for ohnologs, while it is 0 for singletons). The loss of intrinsically disordered regions is discussed further below.

### Evidences for differences in number of intrinsic disorder regions in duplicates

#### Greater change in ohnologs compared to singletons

We have shown above that, since the speciation from *L. kluyveri*, ohnologs have significantly more gain of intrinsically disordered regions when compared to singletons (Figure 1c, Table 3; p = 0.002, Wilcoxon). Similarly, we find that *S. cerevisiae* duplicates, since their divergence from *L. kluyveri*, have significantly more loss than the singletons (p = 5.7e-15, Wilcoxon), with an average of 1.22 intrinsically disordered regions per protein in the ohnologs, as opposed to an average of 0.41 for the singletons (Table 3). This is translated into 37% (197) loss for ohnologs in *S. cerevisiae*, and 25% (1011) in singletons (Table 4). In general, there is significantly more change in ohnologs compared to singletons (p<2.2e-16, Wilcoxon). Indeed, the number of intrinsically disordered regions in ohnologs is significantly different from that found in their orthologs in *L. kluyveri* (p = 0.019 for the closest set of ohnologs in terms of the number of intrinsically disordered regions compared to their orthologs in *L. kluyveri*; p<2.2e-16 for the furthest set, Wilcoxon). This is not seen for the singletons, where we do not detect any significant difference from their orthologs in *L. kluyveri* in terms of intrinsic disorder (p-value = 0.91). These results confirm that singletons have less freedom to evolve their intrinsically disordered regions compared to ohnologs.

We note that an important percentage of proteins in *S. cerevisiae* have retained their number of intrinsically disordered regions constant since their speciation from *L. kluyveri*; these constitute 34% and 50% for ohnologs and singletons respectively (Table 4).

#### More loss than gain in intrinsically disordered regions in ohnologs since the speciation from *L. kluyveri*

**Figure 2:**
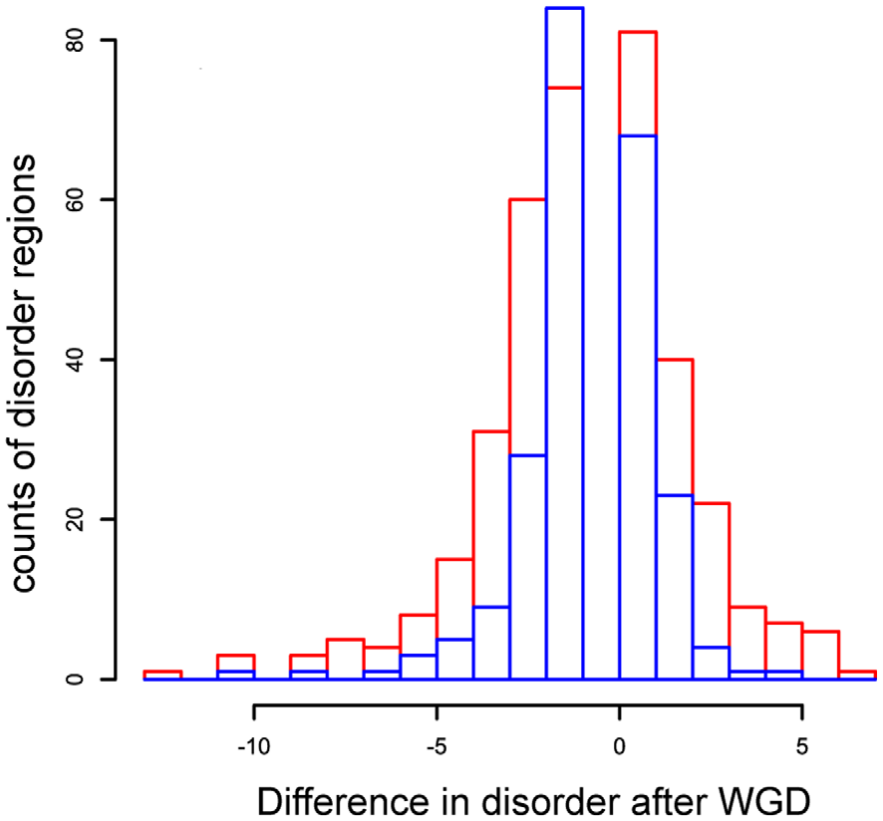
Histogram of the absolute counts of intrinsically disordered regions in *S. cerevisiae* ohnologs after WGD. The two ohnologs of each pair of duplicates are sorted according to them having a closer number of intrinsic disorder regions to their ortholog in *L. kluyveri*. The blue histogram represents the number of intrinsically disordered regions in the ohnolog that is closest in terms of the number of intrinsically disordered regions to its ortholog in *L. kluyveri*; while the red histogram is the one for the furthest ohnolog to its ortholog in *L. kluyveri*.

Table 4 indicates that, when comparing *S. cerevisiae* proteins to their orthologs in *L. kluyveri*, ohnologs tend to lose more intrinsically disordered regions (37%) than gaining them (29%; Table 4, Figure 2, p = 0.011).

Table 4 also indicates that singletons experience the same rate of gain as of loss of intrinsically disordered regions (25.6% and 25.1% respectively). In contrast, the ohnologs have a higher rate of loss than of gain. This observation is consistent with a substantial shift in selection pressures on intrinsically disordered regions following genome duplication, resulting in a net loss of regions of intrinsic disorder on average. We also considered the sets of closest and furthest ohnologs to their ortholog in *L. kluyveri* in terms of the number of intrinsically disordered regions. Table 5 shows that we have more loss than gain in both the closest and furthest sets (25% loss versus 18.4% gain for the closest set; and 38.6% versus 31.4% for the furthest set).

**Table 5:** Comparison of the percentage of intrinsically disordered regions in the ohnologs and singletons.

	Closest ohnolog	Furthest ohnolog	Singleton
Proteins with identical numbers of intrinsically disordered sequences compared to their ortholog in *L. kluyveri*	56.6% (299)	29.9% (158)	49.3% (1981)
Proteins with more intrinsically disordered regions than their ortholog in *L. kluyveri*	18.4% (97)	31.4% (155)	25.6% (1027)
Proteins with less intrinsically disordered regions than their ortholog in *L. kluyveri*	25.0% (132)	38.6 (204)	25.1% (1011)

The ohnologs were classified depending on them having a closer or less similar number of intrinsically disordered regions, compared to their ortholog in *L. kluyveri*.

Put together, these results show an important difference in the number of intrinsically disordered regions between the ohnologs (after speciation from *L. kluyveri* and WGD), but also between the ohnologs and their *L. kluyveri* ortholog as compared to the singletons and their *L. kluyveri* ortholog.

#### Sequence gain, loss, and conservation and how it might relate to functional differences between ohnologs

**Figure 3:**
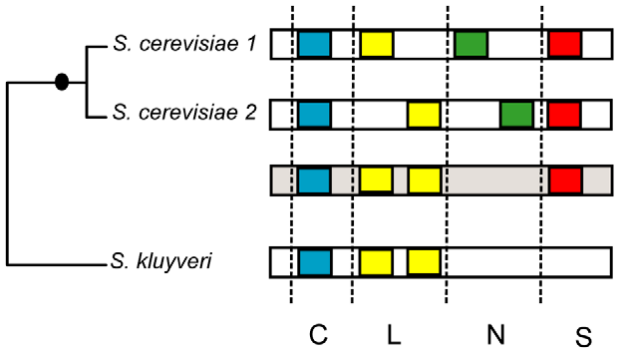
Different scenarios describing the various outcomes of intrinsically disordered regions after WGD in yeast. The relation between *S. cerevisiae* and *L. kluyveri* is represented in the tree on the left of the figure, where the black circle on the tree represents the WGD. Black long rectangles represent a protein that has duplicated in *S. cerevisiae* as a consequence of WGD, and its ortholog in *L. kluyveri*. Intrinsically disordered regions are represented with colored boxes within the rectangles. The grey long rectangle is the orthologous protein in the ancestor that existed prior to WGD and after the speciation from *L. kluyveri*. The vertical dashed lines separate the different scenarios that can affect a intrinsically disordered region after duplication. The first case represented by the blues boxes is a conservation scenario (C, see methods). The yellow boxes represent a loss scenario where one copy of *S. cerevisiae* conserves the intrinsically disordered region, while the other copy loses it (L). The green boxes represent a gain scenario, or the creation of a new intrinsically disordered region in one of the two copies (N). Finally the red represents a creation of an intrinsically disordered region after the divergence from *L. kluyveri* and before WGD (S).

To tease apart more clearly gain, loss, and repartition of intrinsically disordered regions between orthologs, we established four groups of intrinsically disordered regions, based on the appearance or disappearance of at least 30% of this region in the sequence alignments (see Methods). “Gained” regions of intrinsic disorder (Fig. 3, green) are new regions that only exist in one ohnolog; “Lost” regions of intrinsic disorder (Fig. 3, yellow) exist in only one ohnolog and are also present in the *L. kluyveri* ortholog; while “Conserved” regions (Fig. 3, blue) are ones that are found in both ohnologs and in the *L. kluyveri* ortholog. Finally, “Speciation” regions (Fig. 3, red) are new regions found only in both ohnologs and not in the *L. kluyveri* ortholog, these have been most likely created after the speciation from *L. kluyveri* but prior to WGD, or were simply lost in *L. kluyveri*. This approach will misclassify some regions as a result of multiple mutation events, but the overall numbers provide a useful indication of the likely processes of change. The method estimates a lower numbers of cases of gained, lost, or conserved regions of intrinsic disorder (Table 6) compared to the counting method represented in Table 4. This is because in considering presence or absence, it more stringently detects the precise localization of a homologous intrinsically disordered region (see Methods), thus giving greater insights in terms of detecting putative gain and loss of intrinsically disordered regions (Table 6).

**Table 6:** Numbers of intrinsically disordered regions in the ohnologs, binned according to each of the four scenarios represented in Figure 1.

	Gain (of a intrinsically disordered region in one ohnolog compared to *L. kluyveri*)	Speciation (Gain after speciation from *L. kluyveri* and before WGD)	Loss (of a intrinsically disordered region in one ohnolog compared to *L. kluyveri*)	Conservation ( in both ohnologs and in *L. kluyveri*)
Number of intrinsically disordered sequences	177	104	209	817
Percentage of intrinsically disordered sequences	13.54	7.96	15.99	62.51
Number of ohnologs	128	79	117	388
Percentage of ohnologs	27.77	17.14	25.38	84.16

Each region was identified as one of the scenarios using an alignment and gap search (see Methods).

**Figure 4:**
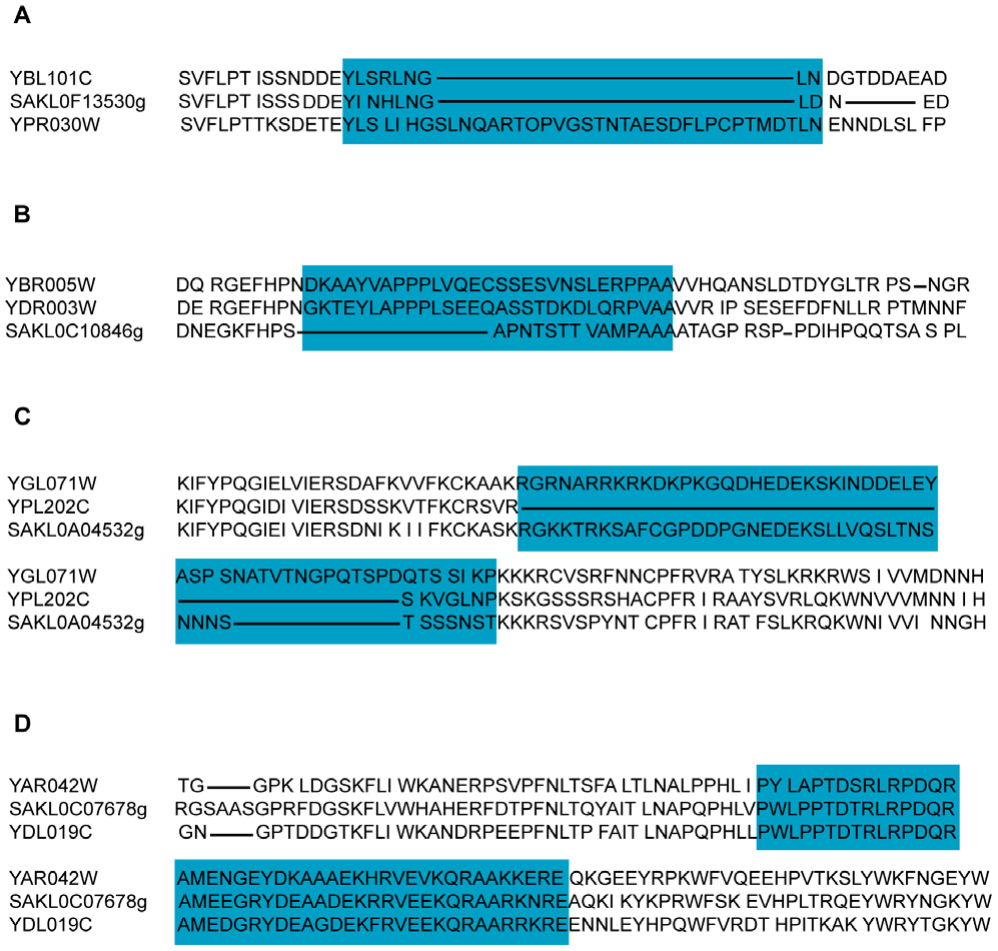
Real examples of proteins undergoing the four scenarios described in **Figure 1**. Blue: intrinsically disordered regions under consideration. (**A**) New region (Category N on Figure 1) of intrinsic disorder in YPR030W aligned with its ohnolog and its ortholog in *L. kluyveri*. (**B**) Creation of intrinsically disordered regions before WGD and after the speciation from *L. kluyveri* in both duplicates YDR003W and YBR005W (scenario S, Figure 1). (**C**) Loss of an intrinsically disordered region in one ohnolog YPL202C (scenario L, Figure 1). (**D**) Conservation of an intrinsically disordered region in both ohnologs and their ortholog in *L. kluyveri* (scenario C, Figure 1).

The majority of intrinsically disordered regions are conserved between the ohnologs and their ortholog in *L. kluyveri* (Table 6, last column). Our results suggest that 13.5% of intrinsically disordered regions have been newly created, and as a consequence may create a new function within the ohnolog (Figure 4a). We detected this scenario in 27.8% of pairs of ohnologs (Table 6; Table S1). 8.0% of the intrinsically disordered regions (termed “Speciation” regions) have been created most likely prior to WGD but after the speciation from L. kluyveri (Figure 4-b); this is detected in 17.1% of ohnologs (Table 6; Table S1). Another 16.0% are consistent with the intrinsically disordered region being lost in one copy, with one ohnolog containing the intrinsically disordered region, and the other not (Figure 4c); we detect this scenario in 25.4% of pairs (Table 6; Table S1). Finally the highest percentage (62.51%) of intrinsically disordered regions are conserved in both ohnologs and outgroup (Figure 3-d); 84% of ohnolog pairs have experienced this scenario (Table 6; Table S1; the three scenarios in *S. cerevisiae* singletons are represented in Table S2). In summary, this analysis suggests approximately equal rates of gain and loss of intrinsically disordered regions.

### Increase in intrinsic disorder of ohnologs and the correlated increase in physical interactions

The acquisition or loss of intrinsic disorder after WGD might determine part of the evolutionary diversification of ohnologs. For example, it has been shown that intrinsically disordered regions are enriched in binding motifs [51], so differences in the number of intrinsically disordered regions might cause differences in the binding propensities of a protein.

To investigate differences that might be due to differential acquisition and loss of intrinsic disorder between ohnologs, the number of physical interactions for each protein was compared to its ohnolog. We assigned each ohnolog of a pair into two sets: those with the lower, and those with the higher number of intrinsically disordered regions. The set of ohnologs with the higher number of intrinsically disordered regions has significantly more physical interactions (mean 27.4; median = 13) than the set that possesses the lower number of intrinsically disordered regions (mean 21.9; median = 10; Table 7; p-value = 0.003). Two possible explanations can account for this result. The first is that an increase in intrinsic disorder after WGD in one ohnolog and a loss in the other consequently increases the number of physical interactions in one and reduces it in the other. This hypothesis is in agreement with the findings that hub proteins possess more intrinsic disorder than proteins with less interaction [6], and that intrinsically disordered regions are enriched in binding motifs [51]. The second explanation is that the ortholog in *L. kluyveri* carries many of the interactions that have subsequently been lost in one of the copies. In other words we are observing a loss instead of a gain of interactions in the duplicates. Because we do not know the number of physical interactions for *L. kluyveri*, we investigated this using the set of ohnologs in *S. cerevisiae* that are the closest in terms of their number of intrinsically disordered regions to that of their ortholog in *L. kluyveri*. We compared the number of interactions of the closest ohnologs to their orthologs in *L. kluyveri*, and showed that there is a significant difference between the closest ohnologs to *L. kluyveri* and the ohnologs that have the highest number of intrinsically disordered regions (p = 4.03e-10, Wilcoxon), suggesting that it is not a bias due to the enrichment in the *L. kluyveri* ortholog. If that were the case, the interactions of the closest set of ohnologs that best represents that of *L. kluyveri* should be similar to the numbers of interactions of the ohnologs with the highest intrinsic disorder which also have the highest number of intrinsic disorder. We further show that the closest set of ohnologs to *L. kluyveri*, in terms of intrinsic disorder, possesses significantly less interactions than that of the ohnologs with the highest number of intrinsically disordered regions (Table 7; p = 2.01e-10, Wilcoxon). Taken together, these results show an increase in physical interaction associated with enrichment in intrinsic disorder. We wanted to test if this result extends to the sets of proteins that our method associated with the loss and gain of a intrinsic disorder region (Table 6, Table S1). We show that the proteins that have undergone gain (47 ohnologs) in their intrinsically disordered regions have significantly more interactions compared to their ohnologs (p = 0.014). Indeed 31 of those 47 proteins have more interactions in the ohnolog with the highest number of intrinsically disordered regions.

**Table 7:** Means and medians of the number of interacting partners of the ohnologs, either classified according to their number of intrinsically disordered regions, or to them having a closer number of intrinsic disorder regions to their ortholog in *L. kluyveri*.

	More intrinsically disordered ohnologs of each pair	Less intrinsically disordered ohnologs of each pair	Closest ohnologs	Furthest ohnologs
Average number of interactors	27,39	21,89	24,34	24,95
Median	13	10	11	12

46 ohnologs have been shown to have undergone only gain (Table S1), of which 24 have more interactions in the copy with the gained intrinsic disorder region(s). However, this was not significant (p-value = 0.338).

## Discussion

We show that loss and gain of intrinsic disorder after WGD can create differences between ohnologs, and consequently create functional divergence between what were originally two identical copies. Our results show that WGD is a mechanism by which intrinsic disorder can expand by creating new regions, or contract by losing a region that is maintained in the other copy. Thus, it is a mechanism by which proteins appear freer to play with their intrinsic disorder by repartitioning the regions between duplicates. We find evidence highly consistent with gain and loss of intrinsically disordered regions. The duplicates that have the highest intrinsic disorder also have a higher number of protein interactions, suggesting that the functional advantages of increasing intrinsic disorder may be to increase the variety of potential interactions, but also consistent with the corollary, that reducing the number of intrinsically disordered regions acts to make a protein's binding patterns less promiscuous and therefore more specific. Thus, differential gain and loss of intrinsically disordered regions can allow the reconfiguration and rewiring of a protein's network, which in turn creates novelty by changing the interaction repertoire of a protein.

From the results it also appears that gain and loss of gene duplicates is strongly associated with increases and decreases in the intrinsically disordered regions. Whether the divergence of intrinsically disordered regions represents one of the primary causative agents in the retention or loss of duplicate genes remains to be proven; nevertheless, this is an attractive hypothesis, given the speed with which novel protein interactions mediated by intrinsic disorder can be gained or lost by the acquisition or deletion of short motifs over evolutionary time.

The impact of intrinsic disorder in creating novelty in a gene, for example, by allowing new interactions, is a relatively new concept, and has not previously been investigated at the genome level. Our work further highlights the accumulating body of evidence supporting the idea that intrinsic disorder plays a critical role in the evolution of eukaryotic protein function. Our findings indicate that protein intrinsic disorder flux should share the same recognition as other well-known mechanisms of genomic generation of novelty such as regulatory flux, alternative splicing and domain shuffling.

A possible issue with our methodology is an over, or under, estimation of the number of intrinsically disordered regions. For example, some mutations may cause an intrinsically disordered region to be mistakenly predicted as two separate regions, or if they are short enough, to be represented as one, as a consequence altering the number of intrinsically disordered regions in one protein by a single residue replacement. To assess whether our conclusions are sensitive to this, we used a second approach that takes into account the percentage of intrinsic disorder residues in a protein. The results yielded similar findings: we show that the gain in intrinsic disorder for ohnologs is higher than that of singletons (Table 8). Similarly, we find a significantly higher rate of loss in ohnologs compared to singletons (p = 4.2e-11). Finally, we find a much greater change (gain and loss) of the percentage of intrinsic disorder residues in ohnologs than singletons, which conserve similar rates of gain and loss (Table 8).

**Table 8:** Average gain and loss in the percentage of intrinsically disordered residues in *S cerevisiae* ohnologs and singletons, in comparison to *L. kluyveri*.

	ohnologs	singletons
Average percentage of intrinsic disorder gained	+3.48%	+2.43%
Average percentage of intrinsic disorder lost	−9.15%	−2.44%

The gain and loss of interacting partners resulting from differential gain and loss of intrinsically disordered regions may partly reflect gain and loss of Short Linear Motifs (SLiMs). SLiMs, which are usually less than 10 amino acids in length, are typically found in intrinsically disordered regions of a protein [52], and often mediate interactions between proteins [53], [54]. However, analysis of SLiMs is not always straightforward, since many observed motifs are false positives that may not interact with high affinity with the peptide-binding domains, such as SH3 and PDZ, that recognize them. Searches can be refined by limiting it to proteins known to interact with specific peptide-binding domains, but protein interaction datasets are highly incomplete and also somewhat error prone. Nevertheless, future studies of the gain and loss of SLiMs following WGD will shed light on the mechanism of generation of these SLiMs, and might also contribute to their annotation by comparing the loss and gain of a SLiM in different ohnologs and the consequences on the protein's binding partners. In addition, predictions of interaction sites additional to SLiMs in intrinsically disordered regions (e.g. alpha-MORFs [55]) may shed further light on the relationship between changes in interactions and the precise sequence regions responsible for these changes.

The differences in the number of interacting partners resulting from differential gain and loss of intrinsically disordered regions in ohnologs are a strong sign of functional specialization between both ohnologs. So far, the best other indicators of functional divergence are alternative spliced isoforms [56] or expression level differences of both duplicates [57]. It will be of great interest in future studies to integrate findings from gene expression divergence, interaction divergence, and disorder divergence. For example, proteins with increased disorder may be expressed at lower levels or for shorter periods [58]. However, expression and splicing analysis are dependent on exposure of the organism to the appropriate conditions, which are not always known. In contrast, intrinsic disorder may be easily evaluated from the available protein sequence, making it easier to quantify functional divergence following gene duplication.

## Methods

### Data

We extracted the sets of ohnologs and singletons in *S. cerevisiae* and their orthologous proteins in *L. kluyveri* from the work of Gordon et al. [59].

We used the Yeast Genome Database (http://www.yeastgenome.org/) to extract the numbers of physical interactions per *S. cerevisiae* protein. Statistical analyses were performed with R.

### Detection of intrinsically disordered sequences

We used IUPred, a free downloadable software for intrinsic disorder detection, with *S. cerevisiae* and *L. kluyveri* proteins [24], [26]. Its algorithm favors the identification of unstructured sequences that do not have the capacity to form sufficient inter-residue interactions to stabilize the polypeptide. IUPred scores each residue of the protein with a value between 0 and 1, depending on its likelihood of being intrinsically disordered. We used the ‘long intrinsic disorder’ prediction parameter that takes into account 100 neighbor residues for the calculation of the intrinsic disorder score. We considered as intrinsically disordered any residue with a score of 0.5 or more.

This setting of IUPred is likely to miss a substantial proportion (approximately a third) of disorder regions [26]. But overall the method has good sensitivity [22], [26], [60]. While other disorder methods may provide somewhat better performance, this method was appropriate for local computation of disorder of many sequences. Choosing lower cut-offs with IUPred or other software may have increased the overall performance in terms of identifying more disordered regions, but would have the unfortunate disadvantage of increasing error arising from false positive identifications.

We defined a region of intrinsic disorder as a sequence of at least 10 consecutive intrinsically disordered amino acids. We initially considered 30 consecutive amino acids, which yielded similar results, but for sample size reasons we extended this threshold to include all intrinsically disordered regions with at least 10 consecutive intrinsically disordered amino acids.

The predictions in our study are based on the use of IUPred. There are limitations in the use of only one prediction tool. Some regions maybe mistakenly predicted as being intrinsically disordered or not. However we think that using another tool will not change the main findings. For example the significant differences between the number of physical interactions for ohnologs with the least intrinsic disorder versus ones with the highest is likely to be independent of the intrinsic disorder prediction tools used.

### Counting and sorting of the number of intrinsically disordered regions per protein

We counted the number of intrinsically disordered regions for each protein belonging to the triplet (ohnolog1, ohnolog2, ortholog in *L. kluyveri*), and for those belonging to the duet (singleton, ortholog in *L. kluyveri*).

**Figure 5:**
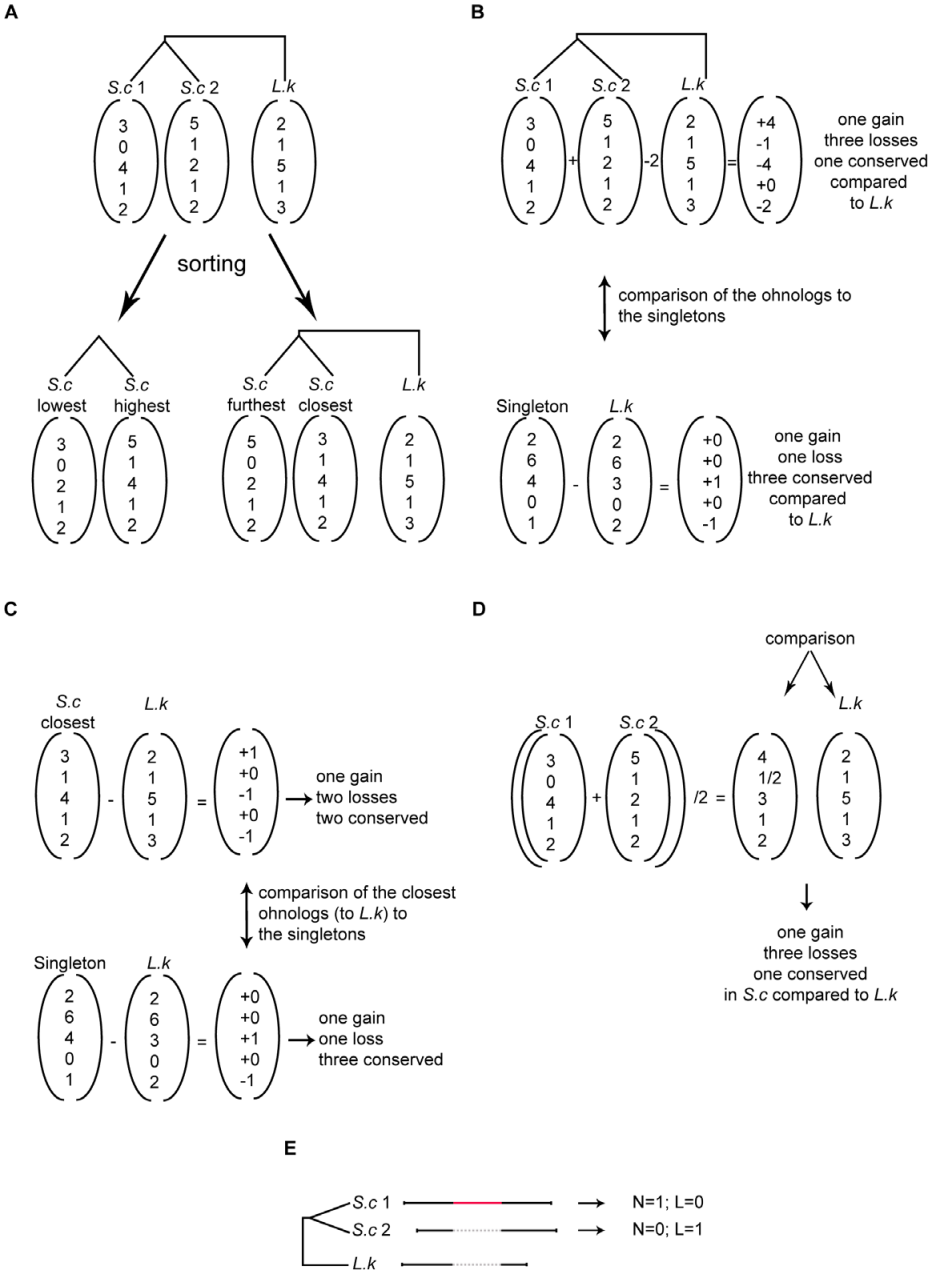
Sorting and comparing the ohnologs and singletons to their ortholog in *L. kluyveri* using an illustrative example. (A) Two distinct ways for sorting the ohnologs. The first is by grouping the ohnologs into highest and lowest according to their number of intrinsically disordered regions. The second is by sorting to ohnologs according to the closest and furthest from the number of intrinsically disordered regions found in their *L. kluyveri* orthologs. (B) Detecting the percentage of gain and loss of intrinsically disordered regions in *S. cerevisiae* compared to *L. kluyveri*. For the singletons, this operation is a simple subtraction from the number of intrinsically disordered regions in *L. kluyveri*. Because we have two ohnologs we need to subtract twice the amount of intrinsically disordered regions in *L. kluyveri* (because both ohnologs were identical at birth, and to consider both ohnologs and singletons equally). (C) Comparison of the gain, loss, and conservation of *S. cerevisiae* closest ohnolog (as defined in figure 5-A) to the gain, loss, and conservation of the singletons in terms of intrinsically disordered region numbers. (D) Detecting gain, loss, and conservation between ohnologs. Because both ohnologs had identical intrinsically disordered region numbers at birth, we do not want to consider twice this number, thus we divide by two. The result from this is then compared to the number of intrinsically disordered regions in *L. kluyveri*. (E) This panel illustrates how we define scenario L (Loss), and scenario N (new intrinsically disordered region) in a protein. The red line in the protein defines an intrinsically disordered region, while the black lines define ordered regions. Grey dashed lines represent gaps in the sequence alignment. For example, a protein has gained a new region and did not loose any, if it satisfies N = 1, and L = 0.

We sorted the two sets of ohnologs according to their intrinsic disorder in two distinct ways (Figure 5-a). First, we systematically arranged the ohnologs in two vectors, one with the highest number of intrinsically disordered regions and the second with the lowest (Figure 5-a). Secondly, we took into account the number of intrinsically disordered regions in the common ortholog of the ohnologs in *L. kluyveri*, and we arranged the ohnologs in two vectors, based on them having a more similar number of intrinsically disordered regions to their ortholog in *L. kluyveri* (Figure 5-a). One vector contained ohnologs that have the closest number of intrinsically disordered regions to that found in their ortholog in *L. kluyveri*, while the second vector contains those that have the furthest number of intrinsically disordered regions.

In addition, we also applied the same methodology for the classification of ohnologs as above, but considering the percentage of intrinsic disorder over the length of the protein instead of the number of intrinsically disordered regions.

### Detecting the percentage of creation and loss of intrinsic disorder after WGD

Because ohnologs and singletons have different degrees of intrinsic disorder even in the ortholog *L. kluyveri* (see Results), we needed a clearer way to compare ohnologs and singletons in terms of their gain and loss of regions, rather than simply their total degree of intrinsic disorder. Accordingly, we compared them in terms of the number of gains and losses of intrinsically disordered regions since the WGD (Figure 5-b). To do this, we added the number of intrinsically disordered regions in the two ohnologs together, and subtracted twice the number found in *L. kluyveri* (hypothesising that the ohnologs possessed an identical number to *L. kluyveri* at birth). For each singleton, we subtracted the number of intrinsically disordered regions from that found for its ortholog in *L. kluyveri* (Figure 5-b). The total number of newly created intrinsically disordered regions for ohnologs was estimated as the sum of all positive values, and the number of lost regions was estimated as the sum of all negative values (Figure 5-b).

### Comparing the closest set and the singletons

We wished to determine whether the set of ohnologs that were most similar to the outgroup in terms of intrinsic disorder, were more similar, or less so, than the singletons. To do this, we compared two vectors, the first containing the differences in the number of intrinsically disordered regions between the closest ohnologs and their orthologs in *L. kluyveri* (Figure 5-c), and the second vector containing the differences between the singletons and their ortholog in *L. kluyveri* (Figure 5-c).

### Finding potential cases of loss of intrinsic disorder regions between the two ohnologs

Here we define loss of intrinsically disordered regions between ohnologs as the reduction or partitioning of the number of intrinsically disordered regions in or between both ohnologs compared to *L. kluyveri*. To determine potential loss cases, we add the number of intrinsically disordered regions of both ohnologs in *S. cerevisiae* and divide this number by two (Figure 5-d). The division by two allows us to put the ohnologs in *S. cerevisiae* on equal footing to their ortholog in *L. kluyveri* (and not counting them twice). We counted the number of cases where this result gave exactly the same number of intrinsically disordered regions when compared to *L. kluyveri*, the cases where it gave more intrinsically disordered regions, and the cases where it gave a lower number of intrinsically disordered regions (Figure 5-d).

### Gain, loss, and conservation of intrinsically disordered regions

We used ClustalW to align the sequences of each triplet *S. cerevisiae*-ohnolog1, *S. cerevisiae*-ohnolog2, and *L. kluyveri* ortholog. We selected blocks in the alignments corresponding to intrinsic disorder in *S. cerevisiae*. We classified the blocks into four categories. Category N (new regions) corresponds to regions of intrinsic disorder in one *S. cerevisiae* ohnolog that are not detected in the other ohnolog, nor in the ortholog in *L. kluyveri* (Figure 3; Figure 5-e). In alignment terms, this correspond to regions in one ohnolog that are aligned with at least 30% gaps compared to both the other ohnolog and the outgroup in *L. kluyveri*. We considered these as typically a new intrinsically disordered region in one copy of the ohnologs. Category S (speciation) corresponds to regions of intrinsic disorder present in both *S. cerevisiae* ohnologs and absent from the *L. kluyveri* ortholog (Figure 3). In terms of alignments this corresponds to a region in one ohnolog that is aligned to a region in the second ohnolog with less than 30% gaps, and more than 30% gaps in their ortholog in *L. kluyveri*. Category L (loss) corresponds to regions of intrinsic disorder present in either of the *S. cerevisiae* ohnologs that are not present in the second ohnolog, but are present in the *L. kluyveri* ortholog (Figure 3; Figure 5-e). The alignment of these regions will show 30% or more gaps versus the ohnolog but not with the *L. kluyveri* ortholog. This category is consistent with the loss of a intrinsically disordered region from one ohnolog, with retention in the other.

Finally, category C (conservation) is consistent with conservation, in which the region is present in both ohnologs and in the *L. kluyveri* ortholog (Figure 3). The alignment of these regions will not yield more than 30% gaps versus the ohnologs nor versus the *L. kluyveri* ortholog.

Because some proteins have more than one region of intrinsic disorder, a protein may have multiple scores. To help sift through this, we sorted the proteins according to the presence of only one type of category, or a combination of the categories C, L, N, or S.

From the above, we define a protein as having acquired a new intrinsically disordered region if it is assigned a score N, but not a score L. We define a protein as having lost a intrinsically disordered region if it is assigned a score L, but not a score N.

We contrasted the number of interaction partners and of intrinsically disordered sequences for putative gain versus putative loss proteins, and compared these to the number seen for their ohnologs. We also counted in each group the number of duplicates that had more interactions and/or more intrinsic disorder than their ohnolog.

### Physical interactions and intrinsic disorder

Using the *Saccharomyces cerevisiae* Genome Database (SGD), we downloaded the number of interactions of each protein of *S. cerevisiae*. First, we tested among ohnologs whether highly intrinsically disordered proteins (that have a higher number of intrinsically disordered regions than their ohnolog) versus lowly intrinsically disordered proteins (that have a lower number of intrinsically disordered regions than their ohnolog) had different numbers of interactions. Secondly, we investigated whether the ohnolog with the greater number of interactors had a different number of intrinsically disordered regions. Finally, we took the set of ohnologs that were most similar to the outgroup in terms of their number of intrinsically disordered regions, and we tested whether, in comparison to the set of other ohnologs, they had a greater number of interactors.

## Supporting Information

Table S1 **Listing of the ohnologs in** ***S. cerevisiae*** **, their orthologs in** ***L. kluyveri*** **, and their corresponding intrinsically disordered regions classified into the four scenarios represented in** **Figure 1** **.** Proteins that have undergone only one scenario, for example a gain of a new intrinsically disordered region, have 1 or more intrinsically disordered region in Category N, and 0's in the other three scenarios.
(PDF)

Table S2 **Listing of the singletons in** ***S. cerevisiae*** **, their orthologs in** ***L. kluyveri*** **, and their corresponding intrinsically disordered regions classified into the three scenarios represented in** **figure 1** **.** Proteins that have undergone only one scenario, for example a gain of a new intrinsically disordered region, have 1 or more intrinsic disorder in scenario, and 0's in the other two scenarios.
(PDF)
