# Supplementary material for: Differences in the Number of Intrinsically Disordered Regions between Yeast Duplicated Proteins, and Their Relationship with Functional Divergence
Source: PLoS One. 2011 Sep 15;6(9):e24989. doi: 10.1371/journal.pone.0024989 (PMC3174238; doi:10.1371/journal.pone.0024989)
Supplement: Table S1 — Listing of the ohnologs in S. cerevisiae , their orthologs in L. kluyveri , and their corresponding intrinsically disordered regions classified into the four scenarios represented in Figure 1 . Proteins that have undergone only one scenario, for example a gain of a new intrinsically disordered region, have 1 or more intrinsically disordered region in Category N, and 0's in the other three scenarios. (PDF) [file pone.0024989.s001.pdf]

ID *S. c* ohnolog1	ID *S. c* ohnolog2	ID *L. k* ancestor	Putative New disorder regions (Scenario N)	Number of disorder regions that have been created prior WGD after the Speciation from *L. k* (Scenario S)	Putative Lost disorder regions (Scenario L)	Number of disorder regions that are conserved between *S. c*, and *L. k* (Scenario C)
YFR040W	YGL229C	SAKL0F01320g	0	0	2	0
YNL074C	YIL135C	SAKL0E09218g	0	2	0	6
YGL133W	YPL216W	SAKL0A04004g	1	1	2	2
YER067W	YIL057C	SAKL0F08822g	0	0	0	1
YAL056W	YOR371C	SAKL0D14740g	0	0	0	5
YHL034C	YLL046C	SAKL0H26004g	0	0	0	4
YGL021W	YBL009W	SAKL0H21648g	1	1	0	2
YOR317W	YMR246W	SAKL0B06094g	0	0	0	1
YGR162W	YGL049C	SAKL0H23694g	0	0	0	2
YOR109W	YNL106C	SAKL0E10494g	0	0	0	2
YGR097W	YPR115W	SAKL0F06314g	2	0	1	4
YMR102C	YKL121W	SAKL0E03520g	0	0	0	2
YLR183C	YDR501W	SAKL0C01936g	0	0	0	1
YOL043C	YAL015C	SAKL0B09790g	0	1	0	0
YHR152W	YGR230W	SAKL0G15488g	0	1	0	1
YDR348C	YHR097C	SAKL0G07568g	0	0	0	2
YGL060W	YBR216C	SAKL0A07194g	0	0	0	1
YNL058C	YIL117C	SAKL0E08536g	0	0	0	2
YNR002C	YCR010C	SAKL0D09328g	0	0	0	1
YPR008W	YGL166W	SAKL0B03344g	1	0	1	0
YHR080C	YDR326C	SAKL0G08404g	1	0	0	4
YMR086W	YKL105C	SAKL0E02926g	0	0	0	2
YBR050C	YDR028C	SAKL0D03762g	0	0	0	1
YLR373C	YGR071C	SAKL0H03916g	1	0	0	0
YDR213W	YLR228C	SAKL0H13024g	2	0	0	3
YER054C	YIL045W	SAKL0F08382g	0	3	0	1
YMR273C	YML109W	SAKL0D01716g	0	0	0	3
YMR295C	YGR273C	SAKL0H01166g	0	0	1	2
YJL065C	YBR278W	SAKL0D07128g	0	0	1	1
YMR031C	YKL050C	SAKL0B11154g	0	0	0	9
YMR285C	YML118W	SAKL0D01166g	0	0	0	3
YER096W	YBL061C	SAKL0F12276g	1	0	0	2
YLR228C	YDR213W	SAKL0H13024g	1	1	0	2
YBR014C	YDL010W	SAKL0C10494g	0	0	0	1
YPR138C	YGR121C	SAKL0F03278g	0	0	0	1
YDL222C	YNL194C	SAKL0E13288g	0	0	0	2
YKL166C	YJL164C	SAKL0C05368g	0	0	0	1
YOR316C	YMR243C	SAKL0B06006g	0	0	0	2
YNL194C	YDL222C	SAKL0E13288g	0	1	0	0
YDL214C	YNL183C	SAKL0E12958g	0	0	0	2
YOR324C	YAL028W	SAKL0B10252g	2	0	0	4
YER045C	YIL036W	SAKL0F07898g	2	0	1	3
YPL105C	YBR172C	SAKL0H08888g	0	0	1	4
YEL063C	YNL270C	SAKL0C02662g	0	1	0	1
YMR183C	YPL232W	SAKL0A03432g	0	0	0	4
YIL151C	YKR096W	SAKL0E15004g	0	0	0	1
YNL065W	YIL120W	SAKL0E08756g	1	0	0	2
YML075C	YLR450W	SAKL0G17886g	0	0	1	1
YLR273C	YOR178C	SAKL0A08184g	0	0	0	4
YJL083W	YKR019C	SAKL0D05808g	1	0	0	1
YNR016C	YMR207C	SAKL0A02046g	0	0	2	2
YDL224C	YNL197C	SAKL0E13354g	0	0	0	7
YKL062W	YMR037C	SAKL0B11330g	0	0	0	4
YHR066W	YDR312W	SAKL0G09306g	0	0	0	2
YGL071W	YPL202C	SAKL0A04532g	1	0	7	4
YHR160C	YGR239C	SAKL0H02574g	0	0	0	1
YPL219W	YGL134W	SAKL0A03938g	0	0	0	2
YBL061C	YER096W	SAKL0F12276g	0	0	1	0
YPL228W	YMR180C	SAKL0A03520g	0	0	0	2
YGR041W	YLR353W	SAKL0B04532g	0	0	0	1
YLR072W	YFL042C	SAKL0B00770g	0	0	0	1
YNL096C	YOR096W	SAKL0E10098g	0	0	0	1
YPL119C	YOR204W	SAKL0H08162g	0	0	0	3
YJL026W	YGR180C	SAKL0G13464g	0	0	1	0
YIL056W	YER064C	SAKL0F08734g	1	0	1	1
YOR019W	YDR475C	SAKL0G06886g	0	0	0	5
YKR078W	YOR069W	SAKL0G00814g	0	0	0	2
YLR108C	YDR132C	SAKL0H16104g	0	0	0	1
YDL070W	YLR399C	SAKL0G15686g	1	1	0	2
YDR508C	YCL025C	SAKL0C01650g	1	0	0	1
YDL211C	YNL176C	SAKL0E12760g	0	0	0	2
YDR273W	YOR042W	SAKL0G01584g	0	0	0	2
YKR077W	YOR066W	SAKL0G00858g	0	0	0	2
YOR033C	YDR263C	SAKL0G07304g	0	0	3	0
YOR371C	YAL056W	SAKL0D14740g	0	0	1	1
YOL100W	YDR490C	SAKL0C09174g	1	0	5	0
YMR124W	YLR031W	SAKL0E04422g	0	0	2	1
YKL008C	YHL003C	SAKL0E02222g	0	0	0	1

YEL030W	YJR045C	SAKL0D11638g	0	0	0	1
YIR033W	YKL020C	SAKL0G19646g	0	1	1	6
YDR043C	YBR066C	SAKL0D03146g	2	1	0	0
YFR022W	YOR018W	SAKL0D09526g	0	0	0	1
YPL049C	YDR480W	SAKL0H11132g	1	0	1	0
YDR003W	YBR005W	SAKL0C10846g	0	1	0	2
YOR110W	YNL108C	SAKL0E10560g	1	0	0	0
YOR076C	YKR084C	SAKL0G00594g	0	0	0	2
YDR312W	YHR066W	SAKL0G09306g	0	0	0	2
YOR342C	YAL037W	SAKL0B10802g	0	0	0	1
YKL043W	YMR016C	SAKL0D13442g	0	0	0	1
YDR522C	YCL048W	SAKL0C00968g	0	0	0	1
YDL048C	YLR375W	SAKL0H03894g	1	2	1	3
YJL105W	YKR029C	SAKL0D05148g	0	0	1	0
YGL166W	YPR008W	SAKL0B03344g	0	0	0	2
YGL197W	YER132C	SAKL0H25212g	0	0	0	2
YOL115W	YNL299W	SAKL0C12430g	0	0	0	1
YML118W	YMR285C	SAKL0D01166g	0	0	0	1
YPL176C	YOR256C	SAKL0H05896g	0	0	0	1
YDR223W	YLR223C	SAKL0H12782g	0	0	0	2
YDL239C	YNL225C	SAKL0E14212g	0	0	4	2
YGR241C	YHR161C	SAKL0H02530g	0	1	0	0
YPL198W	YGL076C	SAKL0A04686g	0	0	0	2
YDR326C	YHR080C	SAKL0G08404g	0	0	0	5
YLR029C	YMR121C	SAKL0E04356g	0	0	0	3
YKL093W	YMR081C	SAKL0E02574g	0	1	0	5
YNR044W	YCR089W	SAKL0A00572g	0	0	0	1
YOR247W	YPL163C	SAKL0H06226g	0	0	0	4
YNR031C	YCR073C	SAKL0A01254g	2	0	1	1
YDR463W	YHR006W	SAKL0G06292g	0	0	0	3
YMR233W	YOR295W	SAKL0B05170g	0	0	0	2
YLL010C	YLR019W	SAKL0G12870g	0	0	0	2
YNL004W	YCL011C	SAKL0D08558g	0	0	0	1
YHR033W	YDR300C	SAKL0G09856g	0	0	0	1
YBL079W	YER105C	SAKL0F12694g	1	1	0	1
YGL107C	YBR238C	SAKL0A06160g	0	0	0	1
YOR173W	YLR270W	SAKL0A08470g	0	0	0	1
YLR136C	YDR151C	SAKL0H15334g	0	0	0	2
YGR056W	YLR357W	SAKL0B04950g	1	0	1	4
YLR260W	YOR171C	SAKL0E02068g	3	1	0	0
YCR010C	YNR002C	SAKL0D09328g	0	0	0	1
YOR086C	YNL087W	SAKL0E09746g	0	1	0	1
YNL309W	YOL131W	SAKL0C12958g	2	0	0	0
YJR127C	YML081W	SAKL0G18062g	1	0	0	6
YKR098C	YIL156W	SAKL0E15136g	0	0	0	2
YPR120C	YGR109C	SAKL0F06688g	0	0	1	1
YOR028C	YDR259C	SAKL0G07128g	1	1	0	4
YDL134C	YDL188C	SAKL0F10164g	0	0	0	2
YDR277C	YOR047C	SAKL0G01474g	1	0	0	0
YLR354C	YGR043C	SAKL0B04642g	0	0	0	1
YDR530C	YCL050C	SAKL0C00792g	0	0	0	1
YPR115W	YGR097W	SAKL0F06314g	0	3	1	7
YBL009W	YGL021W	SAKL0H21648g	0	0	0	5
YPR174C	YLR457C	SAKL0F15906g	0	0	0	1
YLR187W	YNL278W	SAKL0C02222g	0	0	0	3
YNL068C	YIL131C	SAKL0E09020g	3	0	0	4
YDR309C	YHR061C	SAKL0G09526g	0	1	2	2
YML058W	YLR437C	SAKL0G17160g	0	0	1	2
YPL230W	YMR182C	SAKL0A03476g	1	0	1	2
YBL056W	YER089C	SAKL0F12012g	0	0	1	1
YER089C	YBL056W	SAKL0F12012g	0	0	0	1
YDL175C	YIL079C	SAKL0F09636g	0	0	0	2
YPL216W	YGL133W	SAKL0A04004g	0	0	0	1
YOR062C	YKR075C	SAKL0G00968g	0	0	0	1
YDR046C	YBR068C	SAKL0D02970g	0	0	0	2
YDR096W	YER169W	SAKL0H17842g	2	2	1	2
YLR238W	YDR200C	SAKL0H13442g	0	0	0	3
YGR239C	YHR160C	SAKL0H02574g	0	1	0	0
YCR037C	YJL198W	SAKL0C04224g	1	0	0	1
YNL283C	YOL105C	SAKL0C09086g	0	1	0	1
YHR056C	YDR303C	SAKL0G09768g	1	1	0	0
YNL106C	YOR109W	SAKL0E10494g	0	0	1	2
YNL093W	YOR089C	SAKL0E09922g	0	0	0	1
YGL134W	YPL219W	SAKL0A03938g	0	0	0	2
YER150W	YDR077W	SAKL0H18700g	0	0	0	1
YKL201C	YJR061W	SAKL0D12672g	4	1	0	2
YKR003W	YHR001W	SAKL0A08932g	0	0	1	0
YER120W	YBL091C-A	SAKL0F13200g	0	0	1	0
YER132C	YGL197W	SAKL0H25212g	2	0	3	6
YDL109C	YGL144C	SAKL0E06578g	0	0	0	1
YGR143W	YPR159W	SAKL0F02310g	0	0	0	1

YMR037C	YKL062W	SAKL0B11330g	0	3	0	5
YOR066W	YKR077W	SAKL0G00858g	5	0	0	4
YGR221C	YHR149C	SAKL0G15290g	0	1	0	3
YBR197C	YPL077C	SAKL0H09944g	0	0	0	1
YGR197C	YJR015W	SAKL0C13508g	2	0	0	1
YDR409W	YOR156C	SAKL0G04334g	1	0	1	0
YDR505C	YLR177W	SAKL0C01804g	1	0	1	3
YGL144C	YDL109C	SAKL0E06578g	0	0	1	0
YOL016C	YFR014C	SAKL0D07656g	0	0	1	0
YLR133W	YDR147W	SAKL0H15444g	0	0	1	1
YKL105C	YMR086W	SAKL0E02926g	2	1	0	1
YPL163C	YOR247W	SAKL0H06226g	1	0	0	1
YMR047C	YKL068W	SAKL0B11770g	0	0	0	3
YNL293W	YOL112W	SAKL0C12298g	1	0	3	0
YPL177C	YGL096W	SAKL0A05610g	1	0	0	0
YOR227W	YPL137C	SAKL0H07216g	0	1	0	3
YPL026C	YDR247W	SAKL0H11748g	1	0	0	0
YJL099W	YKR027W	SAKL0D05434g	0	0	0	1
YBR049C	YDR026C	SAKL0D03806g	2	1	0	0
YOR295W	YMR233W	SAKL0B05170g	0	0	0	2
YKR028W	YJL098W	SAKL0D05456g	0	1	0	1
YKL035W	YHL012W	SAKL0A09834g	0	0	0	1
YJL048C	YBR273C	SAKL0D06556g	0	0	0	1
YJL129C	YKR050W	SAKL0C06622g	2	0	0	1
YKR084C	YOR076C	SAKL0G00594g	0	1	0	1
YDR001C	YBR001C	SAKL0C11176g	0	0	0	2
YPL137C	YOR227W	SAKL0H07216g	0	0	1	3
YML082W	YJR130C	SAKL0G18172g	1	0	0	1
YCL036W	YDR514C	SAKL0C01276g	0	1	0	0
YIL156W	YKR098C	SAKL0E15136g	0	0	1	0
YJL070C	YBR284W	SAKL0D06380g	0	0	3	1
YLR437C	YML058W	SAKL0G17160g	0	0	1	0
YKR100C	YIL158W	SAKL0E15202g	0	0	4	0
YNR019W	YCR048W	SAKL0A01914g	1	0	0	3
YHR061C	YDR309C	SAKL0G09526g	1	0	0	1
YKR029C	YJL105W	SAKL0D05148g	0	1	1	0
YBR182C	YPL089C	SAKL0H09416g	0	0	0	5
YDR151C	YLR136C	SAKL0H15334g	0	1	1	1
YFR013W	YOL017W	SAKL0D07612g	0	0	1	0
YDR264C	YOR034C	SAKL0G07326g	1	0	0	1
YOL036W	YIR016W	SAKL0F04246g	0	0	7	1
YIL045W	YER054C	SAKL0F08382g	1	2	0	2
YOL028C	YIR018W	SAKL0F05434g	0	0	0	1
YOR326W	YAL029C	SAKL0B10274g	0	0	3	1
YDR490C	YOL100W	SAKL0C09174g	0	0	1	1
YDR304C	YHR057C	SAKL0G09746g	1	0	0	0
YLR450W	YML075C	SAKL0G17886g	0	0	0	1
YNL183C	YDL214C	SAKL0E12958g	1	0	2	1
YLR371W	YGR070W	SAKL0H03960g	1	0	1	0
YLR324W	YGR004W	SAKL0H22902g	0	0	0	1
YBR284W	YJL070C	SAKL0D06380g	0	0	0	1
YPR030W	YBL101C	SAKL0F13530g	2	5	0	2
YDR253C	YPL038W	SAKL0H11440g	1	0	0	0
YPR154W	YGR136W	SAKL0F02486g	0	0	0	1
YDR303C	YHR056C	SAKL0G09768g	2	1	1	0
YNL014W	YLR249W	SAKL0D08316g	0	0	0	3
YKL148C	YJL045W	SAKL0G11440g	0	0	0	3
YGR238C	YHR158C	SAKL0H02618g	0	0	0	9
YPL256C	YMR199W	SAKL0A02508g	1	0	0	1
YOR204W	YPL119C	SAKL0H08162g	0	0	0	4
YHR103W	YDR351W	SAKL0G01870g	0	1	0	2
YNL176C	YDL211C	SAKL0E12760g	4	0	0	3
YKL068W	YMR047C	SAKL0B11770g	0	0	0	1
YDL019C	YAR042W	SAKL0C07678g	0	0	0	2
YPR159W	YGR143W	SAKL0F02310g	0	0	0	2
YIL105C	YNL047C	SAKL0E08162g	0	1	0	2
YGL229C	YFR040W	SAKL0F01320g	1	0	0	1
YDR389W	YOR134W	SAKL0G03476g	0	0	2	0
YBR172C	YPL105C	SAKL0H08888g	1	0	0	4
YNR028W	YCR069W	SAKL0A01364g	0	0	0	1
YER114C	YBL085W	SAKL0F12958g	1	1	1	2
YDL194W	YDL138W	SAKL0F10362g	1	1	0	0
YKR021W	YJL084C	SAKL0D05764g	0	1	0	3
YOR178C	YLR273C	SAKL0A08184g	1	0	0	5
YIL135C	YNL074C	SAKL0E09218g	0	1	0	1
YFL004W	YPL019C	SAKL0B02376g	0	0	0	2
YHR016C	YFR024C-A	SAKL0D09702g	0	0	0	1
YLR277C	YOR179C	SAKL0A08096g	0	0	3	0
YLR457C	YPR174C	SAKL0F15906g	0	0	1	1
YDR358W	YHR108W	SAKL0G02068g	0	0	0	3
YMR195W	YPL250C	SAKL0A02684g	0	0	0	1

YGR023W	YLR332W	SAKL0B03608g	0	1	0	0
YKR089C	YOR081C	SAKL0E14564g	1	0	3	0
YLR206W	YDL161W	SAKL0F10934g	1	0	1	2
YGL228W	YFR039C	SAKL0F01342g	0	0	2	2
YOL103W	YDR497C	SAKL0C09350g	0	1	0	0
YGL139W	YPL221W	SAKL0A03828g	0	0	0	1
YOR193W	YPL112C	SAKL0H08492g	0	0	0	1
YPL229W	YMR181C	SAKL0A03498g	2	2	0	0
YJL084C	YKR021W	SAKL0D05764g	0	0	1	1
YOR054C	YKR072C	SAKL0G01166g	0	0	1	4
YBR278W	YJL065C	SAKL0D07128g	1	0	0	1
YCL024W	YDR507C	SAKL0C01716g	0	0	0	2
YOR018W	YFR022W	SAKL0D09526g	0	0	1	2
YKR036C	YJL112W	SAKL0D04796g	0	0	0	1
YLR433C	YML057W	SAKL0G17094g	0	0	0	1
YLR353W	YGR041W	SAKL0B04532g	0	0	0	2
YER059W	YIL050W	SAKL0F08602g	1	0	0	0
YJR030C	YJL181W	SAKL0C04752g	0	0	0	2
YDR259C	YOR028C	SAKL0G07128g	2	0	0	1
YER102W	YBL072C	SAKL0F12540g	0	0	0	1
YPL019C	YFL004W	SAKL0B02376g	1	0	0	2
YIL131C	YNL068C	SAKL0E09020g	0	0	0	1
YPR111W	YGR092W	SAKL0F06094g	0	0	0	2
YDR122W	YLR096W	SAKL0H16632g	0	0	0	1
YDR200C	YLR238W	SAKL0H13442g	2	0	0	2
YER098W	YBL067C	SAKL0F12430g	0	0	1	2
YKR010C	YJL076W	SAKL0D06094g	0	0	0	3
YBR001C	YDR001C	SAKL0C11176g	1	0	0	0
YOR256C	YPL176C	SAKL0H05896g	0	0	1	1
YIL036W	YER045C	SAKL0F07898g	3	1	1	2
YLR332W	YGR023W	SAKL0B03608g	0	0	0	1
YGR043C	YLR354C	SAKL0B04642g	0	0	0	1
YJL110C	YKR034W	SAKL0D04928g	2	1	4	3
YBL085W	YER114C	SAKL0F12958g	0	0	0	2
YAR042W	YDL019C	SAKL0C07678g	0	0	0	4
YOR127W	YDR379W	SAKL0G02926g	1	1	0	5
YBL101C	YPR030W	SAKL0F13530g	0	3	1	6
YPL232W	YMR183C	SAKL0A03432g	0	0	0	2
YDR206W	YLR233C	SAKL0H13288g	0	0	4	1
YKL198C	YJR059W	SAKL0D12628g	0	0	0	3
YPR042C	YJR091C	SAKL0F14102g	0	1	0	2
YDR480W	YPL049C	SAKL0H11132g	0	0	0	2
YLL016W	YLR310C	SAKL0H24222g	1	0	0	1
YBL089W	YER119C	SAKL0F13134g	0	0	0	1
YBL075C	YER103W	SAKL0F12606g	0	0	0	3
YHR108W	YDR358W	SAKL0G02068g	0	0	0	3
YER035W	YGL222C	SAKL0F01628g	0	0	0	1
YMR153W	YDL088C	SAKL0E05566g	1	0	0	1
YCL011C	YNL004W	SAKL0D08558g	0	0	0	3
YMR121C	YLR029C	SAKL0E04356g	0	0	0	3
YDL010W	YBR014C	SAKL0C10494g	0	0	0	1
YDR028C	YBR050C	SAKL0D03762g	2	0	6	0
YKR075C	YOR062C	SAKL0G00968g	1	0	0	1
YHR015W	YFR023W	SAKL0D09680g	0	0	2	0
YHR006W	YDR463W	SAKL0G06292g	1	1	0	3
YNL009W	YLR174W	SAKL0D08426g	0	0	0	1
YAL015C	YOL043C	SAKL0B09790g	0	0	0	1
YOR096W	YNL096C	SAKL0E10098g	0	0	0	1
YIR016W	YOL036W	SAKL0F04246g	0	0	0	1
YLR375W	YDL048C	SAKL0H03894g	0	0	0	3
YDR146C	YLR131C	SAKL0H15488g	0	0	0	7
YHR149C	YGR221C	SAKL0G15290g	0	0	0	7
YBR068C	YDR046C	SAKL0D02970g	1	0	0	1
YMR081C	YKL093W	SAKL0E02574g	0	0	0	2
YOL131W	YNL309W	SAKL0C12958g	0	0	0	2
YML111W	YMR275C	SAKL0D01606g	0	1	0	0
YMR016C	YKL043W	SAKL0D13442g	1	0	2	0
YOL105C	YNL283C	SAKL0C09086g	1	3	0	2
YGL096W	YPL177C	SAKL0A05610g	0	0	0	3
YNL270C	YEL063C	SAKL0C02662g	0	1	0	0
YER129W	YGL179C	SAKL0H24706g	1	0	4	1
YNL157W	YHR132W-A	SAKL0E11968g	0	0	0	1
YDR351W	YHR103W	SAKL0G01870g	0	1	0	6
YDR515W	YCL037C	SAKL0C01254g	0	0	0	1
YOL092W	YBR147W	SAKL0C09856g	0	0	1	0
YBR273C	YJL048C	SAKL0D06556g	0	0	1	1
YAL017W	YOL045W	SAKL0B09856g	2	0	8	2
YMR115W	YKL133C	SAKL0E03916g	0	0	0	1
YMR199W	YPL256C	SAKL0A02508g	1	0	0	3
YKL039W	YHL017W	SAKL0A10032g	0	1	0	0
YIL057C	YER067W	SAKL0F08822g	0	0	0	2

YCL051W	YDR528W	SAKL0C00748g	1	1	2	6
YLR120C	YDR144C	SAKL0H15664g	0	0	1	1
YJL164C	YKL166C	SAKL0C05368g	0	0	0	1
YMR177W	YPL224C	SAKL0A03652g	0	0	0	2
YER101C	YBL069W	SAKL0F12496g	0	0	0	1
YML007W	YDR423C	SAKL0G04950g	2	0	4	2
YBL072C	YER102W	SAKL0F12540g	0	0	0	1
YCR052W	YNR023W	SAKL0A01804g	0	1	0	1
YAL053W	YOR365C	SAKL0D14586g	0	0	0	1
YER144C	YDR069C	SAKL0H18986g	0	0	0	1
YJL013C	YGR188C	SAKL0G13882g	0	0	0	1
YML016C	YDR436W	SAKL0G05390g	1	0	0	0
YER064C	YIL056W	SAKL0F08734g	0	0	0	5
YGR070W	YLR371W	SAKL0H03960g	0	0	0	1
YJL116C	YKR042W	SAKL0C07194g	0	0	0	1
YPL212C	YGL063W	SAKL0A04158g	1	0	0	1
YPL141C	YOR233W	SAKL0H07084g	0	0	1	3
YDR528W	YCL051W	SAKL0C00748g	0	0	0	2
YOR069W	YKR078W	SAKL0G00814g	0	0	0	1
YHR133C	YNL156C	SAKL0E11990g	1	0	0	0
YML081W	YJR127C	SAKL0G18062g	0	1	0	2
YOL017W	YFR013W	SAKL0D07612g	0	0	0	4
YDR507C	YCL024W	SAKL0C01716g	0	0	1	1
YKL027W	YHR003C	SAKL0A09328g	0	0	1	0
YPL202C	YGL071W	SAKL0A04532g	0	0	0	1
YDR458C	YML034W	SAKL0G06116g	0	0	0	1
YDR069C	YER144C	SAKL0H18986g	0	0	2	0
YMR305C	YGR279C	SAKL0H00484g	0	0	0	1
YJR031C	YEL022W	SAKL0D11088g	0	0	0	1
YJL112W	YKR036C	SAKL0D04796g	0	0	2	1
YLR248W	YGL158W	SAKL0D08294g	2	0	1	2
YEL022W	YJR031C	SAKL0D11088g	1	0	0	1
YBL054W	YER088C	SAKL0F11968g	0	2	0	4
YGR273C	YMR295C	SAKL0H01166g	0	0	0	1
YML057W	YLR433C	SAKL0G17094g	1	0	0	2
YBR150C	YOL089C	SAKL0C09944g	2	0	0	1
YKL020C	YIR033W	SAKL0G19646g	0	1	1	6
YNL116W	YHR115C	SAKL0E10824g	1	0	0	0
YER166W	YDR093W	SAKL0H17908g	0	0	0	4
YBL005W	YGL013C	SAKL0D01100g	0	1	0	1
YDR132C	YLR108C	SAKL0H16104g	0	0	1	0
YAL029C	YOR326W	SAKL0B10274g	0	0	0	1
YNL108C	YOR110W	SAKL0E10560g	0	0	0	1
YKL203C	YJR066W	SAKL0D12826g	0	0	0	3
YKL072W	YMR053C	SAKL0B11946g	0	0	0	1
YMR182C	YPL230W	SAKL0A03476g	0	0	0	1
YOL089C	YBR150C	SAKL0C09944g	0	0	0	2
YIL149C	YKR095W	SAKL0E14916g	0	0	0	6
YOR171C	YLR260W	SAKL0E02068g	0	3	0	0
YIL050W	YER059W	SAKL0F08602g	0	0	0	1
YJR091C	YPR042C	SAKL0F14102g	0	0	0	3
YOL091W	YBR148W	SAKL0C09878g	0	0	0	2
YKR042W	YJL116C	SAKL0C07194g	0	0	1	1
YIL095W	YNL020C	SAKL0E07722g	1	0	0	4
YBR148W	YOL091W	SAKL0C09878g	0	0	1	4
YDR514C	YCL036W	SAKL0C01276g	0	0	0	1
YOR338W	YAL034C	SAKL0B10560g	0	0	0	1
YER088C	YBL054W	SAKL0F11968g	0	0	1	2
YER158C	YDR085C	SAKL0H18282g	0	0	0	2
YER027C	YGL208W	SAKL0F02002g	0	0	0	1
YPR172W	YLR456W	SAKL0F15862g	0	1	0	1
YDR451C	YML027W	SAKL0G05896g	0	0	0	4
YLR096W	YDR122W	SAKL0H16632g	1	0	1	3
YDR475C	YOR019W	SAKL0G06886g	1	0	2	5
YEL060C	YOR003W	SAKL0E01012g	1	0	0	1
YMR243C	YOR316C	SAKL0B06006g	0	0	1	0
YMR264W	YML101C	SAKL0D02090g	0	0	0	1
YPL249C	YMR192W	SAKL0A02816g	0	0	0	2
YNR047W	YCR091W	SAKL0A00484g	0	0	1	1
YLR131C	YDR146C	SAKL0H15488g	1	0	0	6
YBR238C	YGL107C	SAKL0A06160g	2	0	0	1
YNR023W	YCR052W	SAKL0A01804g	0	1	1	0
YLR233C	YDR206W	SAKL0H13288g	0	0	0	3
YPL077C	YBR197C	SAKL0H09944g	0	2	0	0
YDR085C	YER158C	SAKL0H18282g	1	0	0	3
YAL028W	YOR324C	SAKL0B10252g	0	1	0	2
YNL278W	YLR187W	SAKL0C02222g	0	0	0	2
YML027W	YDR451C	SAKL0G05896g	0	0	0	2
YLR313C	YLL021W	SAKL0H20174g	0	0	0	2
YGR188C	YJL013C	SAKL0G13882g	0	0	2	0
YOL045W	YAL017W	SAKL0B09856g	0	0	0	3

YLL046C	YHL034C	SAKL0H26004g	0	0	0	1
YER028C	YGL209W	SAKL0F01980g	0	0	0	4
YLR357W	YGR056W	SAKL0B04950g	1	0	0	2
YOR293W	YMR230W	SAKL0H04356g	0	0	0	1
YLR399C	YDL070W	SAKL0G15686g	0	0	1	0
YJL058C	YBR270C	SAKL0D06842g	0	0	0	4
YDL088C	YMR153W	SAKL0E05566g	1	0	0	3
YLL048C	YHL035C	SAKL0H26026g	0	0	0	1
YBR140C	YOL081W	SAKL0C10340g	1	3	0	2
YNL299W	YOL115W	SAKL0C12430g	1	0	0	0
YGR092W	YPR111W	SAKL0F06094g	0	0	0	2
YER169W	YDR096W	SAKL0H17842g	0	1	0	2
YHR158C	YGR238C	SAKL0H02618g	1	0	1	2
YJL098W	YKR028W	SAKL0D05456g	1	0	0	2
YNL160W	YHR139C	SAKL0E12298g	1	0	0	0
YDR501W	YLR183C	SAKL0C01936g	0	0	1	1
YNL053W	YIL113W	SAKL0E08404g	1	0	3	0
YCR089W	YNR044W	SAKL0A00572g	2	0	5	3
YPR119W	YGR108W	SAKL0F06666g	0	0	0	1
YDR379W	YOR127W	SAKL0G02926g	0	0	0	1
YHR131C	YNL144C	SAKL0E11858g	2	0	0	5
YNL121C	YHR117W	SAKL0E10956g	1	0	0	0
YKR096W	YIL151C	SAKL0E15004g	0	0	1	1
YLR310C	YLL016W	SAKL0H24222g	2	0	6	1
YNL225C	YDL239C	SAKL0E14212g	0	0	0	2
YML065W	YLR442C	SAKL0G17446g	1	0	0	0
YLR223C	YDR223W	SAKL0H12782g	2	0	2	1
YHR115C	YNL116W	SAKL0E10824g	0	0	0	1
YML034W	YDR458C	SAKL0G06116g	0	0	1	1
YFR023W	YHR015W	SAKL0D09680g	0	0	1	1
YML109W	YMR273C	SAKL0D01716g	0	0	2	3
YPR032W	YBL106C	SAKL0F13640g	1	0	0	1
YDL138W	YDL194W	SAKL0F10362g	0	0	0	2
YDR093W	YER166W	SAKL0H17908g	0	0	0	4
YML101C	YMR264W	SAKL0D02090g	0	0	0	2
YCL037C	YDR515W	SAKL0C01254g	0	0	0	2
YHR117W	YNL121C	SAKL0E10956g	1	0	0	0
YOR233W	YPL141C	SAKL0H07084g	0	0	0	3
YOL066C	YDL036C	SAKL0C08668g	0	0	0	1
YGL208W	YER027C	SAKL0F02002g	0	0	0	1
YNL197C	YDL224C	SAKL0E13354g	0	0	0	3
YDR077W	YER150W	SAKL0H18700g	2	0	0	1
YJL165C	YKL168C	SAKL0C05280g	2	0	1	3
YDR252W	YPL037C	SAKL0H11462g	0	0	0	1
YLR350W	YGR038W	SAKL0B04356g	0	1	0	0
YMR230W	YOR293W	SAKL0H04356g	0	0	0	1
YGL209W	YER028C	SAKL0F01980g	0	0	0	5
YLL021W	YLR313C	SAKL0H20174g	0	0	6	1
YMR275C	YML111W	SAKL0D01606g	0	0	1	3
YKL121W	YMR102C	SAKL0E03520g	1	0	0	2
YGL013C	YBL005W	SAKL0D01100g	3	2	0	1
YJL181W	YJR030C	SAKL0C04752g	0	0	0	1
YKL050C	YMR031C	SAKL0B11154g	1	1	0	3
YOL081W	YBR140C	SAKL0C10340g	0	0	1	2
YKR095W	YIL149C	SAKL0E14916g	1	1	2	9
YGR230W	YHR152W	SAKL0G15488g	0	1	0	0
YGR032W	YLR342W	SAKL0B04158g	0	0	0	3
YOR134W	YDR389W	SAKL0G03476g	0	0	0	1
YGR109C	YPR120C	SAKL0F06688g	0	0	0	1
