# Supplementary material for: Differences in the Number of Intrinsically Disordered Regions between Yeast Duplicated Proteins, and Their Relationship with Functional Divergence
Source: PLoS One. 2011 Sep 15;6(9):e24989. doi: 10.1371/journal.pone.0024989 (PMC3174238; doi:10.1371/journal.pone.0024989)
Supplement: Table S2 — Listing of the singletons in S. cerevisiae , their orthologs in L. kluyveri , and their corresponding intrinsically disordered regions classified into the three scenarios represented in figure 1 . Proteins that have undergone only one scenario, for example a gain of a new intrinsically disordered region, have 1 or more intrinsic disorder in scenario, and 0's in the other two scenarios. (PDF) [file pone.0024989.s002.pdf]

ID *S. c* single	ID *L. k*	Putative New disorder regions (Scenario N)	putative Lost disorder regions (Scenario L)	Number of disorder regions that are conserved between *S. c*, and *L. k* (Scenario C)
YDL225W	SAKL0E13376g	0	2	0
YCL029C	SAKL0C01496g	0	1	0
YAL047C	SAKL0D14454g	0	3	0
YJL176C	SAKL0C04884g	0	1	0
YNL243W	SAKL0C03652g	0	2	0
YPL064C	SAKL0H10318g	0	1	0
YPR067W	SAKL0B07744g	0	1	0
YKL145W	SAKL0G11484g	0	0	5
YNL264C	SAKL0C02882g	0	1	0
YHR136C	SAKL0E12144g	0	2	0
YLR274W	SAKL0A08162g	0	2	0
YJL056C	SAKL0D06776g	0	1	0
YGR103W	SAKL0F06512g	0	5	0
YGL219C	SAKL0F01716g	0	3	0
YBR045C	SAKL0D03883g	0	2	0
YMR312W	SAKL0H00880g	0	1	0
YIL047C	SAKL0F08448g	0	0	2
YLR141W	SAKL0H14982g	0	4	0
YDR410C	SAKL0G04356g	0	1	0
YBR162C	SAKL0H10692g	0	1	0
YCR065W	SAKL0A01496g	0	2	0
YLL006W	SAKL0G12716g	0	2	0
YPR091C	SAKL0B08844g	0	2	0
YHR073W	SAKL0G08756g	1	1	5
YBR188C	SAKL0H09702g	0	2	0
YDR386W	SAKL0G03410g	0	1	0
YLR422W	SAKL0G16610g	0	1	0
YKL096W-A	SAKL0E02728g	0	1	0
YKL024C	SAKL0G19756g	0	1	0
YGL251C	SAKL0F00528g	0	3	0
YJL161W	SAKL0C05522g	0	1	0
YDR524C	SAKL0C00924g	0	2	1
YHR079C	SAKL0G08448g	0	1	0
YLR006C	SAKL0G12100g	0	1	0
YFR031C	SAKL0F07282g	0	0	3
YCR035C	SAKL0C04290g	0	1	0
YLL027W	SAKL0H25454g	0	1	0
YFL041W	SAKL0B00836g	0	0	1
YMR291W	SAKL0H01364g	0	1	0
YPL192C	SAKL0A04928g	0	1	0
YCR028C	SAKL0C04708g	0	1	0
YIL159W	SAKL0E15224g	0	1	0
YER058W	SAKL0F08580g	0	1	0
YML061C	SAKL0G17336g	0	1	0
YDR246W	SAKL0H11792g	1	0	1
YJL154C	SAKL0C05764g	0	1	0
YMR029C	SAKL0D13970g	0	2	0
YBR252W	SAKL0F06974g	0	1	0
YMR001C	SAKL0E01584g	0	0	1
YBR081C	SAKL0B06468g	0	1	0
YMR148W	SAKL0E05434g	0	2	0
YML013W	SAKL0G05280g	0	1	0

YGR217W	SAKL0G15114g	0	2	0
YLR066W	SAKL0B00924g	0	1	0
YOR144C	SAKL0G03718g	0	3	0
YJR078W	SAKL0F13684g	0	1	0
YJL123C	SAKL0C06886g	0	1	0
YER075C	SAKL0F09196g	0	2	0
YBR125C	SAKL0B08448g	0	1	0
YLR018C	SAKL0G12848g	0	2	0
YNL253W	SAKL0C03344g	0	1	0
YNR052C	SAKL0A00242g	0	1	0
YLR001C	SAKL0G12496g	0	2	0
YAL026C	SAKL0B10186g	1	1	2
YAL013W	SAKL0B09680g	0	2	0
YBL008W	SAKL0H21846g	1	0	2
YJL197W	SAKL0C04246g	0	4	0
YNL201C	SAKL0E13442g	0	2	0
YMR123W	SAKL0E04400g	0	1	0
YNL115C	SAKL0E10802g	0	1	0
YGL006W	SAKL0H22286g	0	3	2
YOL001W	SAKL0E01408g	0	2	0
YPR160W	SAKL0F02222g	0	1	1
YOR032C	SAKL0G07282g	0	3	0
YCL026C-A	SAKL0C01628g	0	2	0
YJL210W	SAKL0C03762g	0	1	0
YGR229C	SAKL0G15466g	0	1	0
YOL070C	SAKL0C08514g	0	1	0
YBL052C	SAKL0F11946g	0	1	0
YNL152W	SAKL0E11638g	0	1	0
YBL091C	SAKL0F13178g	0	0	1
YER145C	SAKL0H18920g	1	0	0
YLR052W	SAKL0B01760g	0	1	0
YBR205W	SAKL0H10494g	0	1	0
YIR002C	SAKL0F04532g	0	1	0
YMR294W	SAKL0H01232g	0	0	2
YPR086W	SAKL0F04642g	0	1	0
YJR119C	SAKL0F15290g	0	2	0
YHL015W	SAKL0A09966g	0	0	1
YGR236C	SAKL0H02684g	0	1	0
YLR149C	SAKL0H14652g	2	0	4
YJL092W	SAKL0D05588g	0	1	0
YDR363W	SAKL0G02266g	1	1	1
YDR308C	SAKL0G09570g	0	1	0
YHR052W	SAKL0G10890g	1	1	1
YGL017W	SAKL0H21758g	0	1	0
YBR200W	SAKL0H10098g	1	2	2
YBR035C	SAKL0D04400g	0	1	0
YCR002C	SAKL0D08844g	0	1	0
YJL207C	SAKL0C03828g	0	4	0
YLR286C	SAKL0A07964g	0	1	0
YKL155C	SAKL0G11198g	0	3	0
YOR361C	SAKL0D14432g	0	1	0
YOR008C	SAKL0F05412g	1	0	4
YPR084W	SAKL0B08558g	0	2	0

YLR189C	SAKL0C02288g	0	3	0
YOR006C	SAKL0E01144g	0	1	0
YNL277W	SAKL0C02266g	0	0	1
YCL008C	SAKL0D09086g	0	1	0
YBR023C	SAKL0C11726g	0	2	0
YDR182W	SAKL0H13992g	0	0	1
YLR345W	SAKL0B04268g	0	2	0
YIR025W	SAKL0G19580g	0	2	0
YGL022W	SAKL0H21604g	0	1	0
YAL019W	SAKL0B09988g	2	0	4
YJR134C	SAKL0G18414g	0	1	0
YDL114W	SAKL0E06732g	1	1	0
YKL089W	SAKL0E02354g	0	1	0
YIL097W	SAKL0E07854g	0	1	0
YHR050W	SAKL0G10802g	2	0	1
YIL146C	SAKL0E14850g	0	1	0
YLR102C	SAKL0H16324g	0	1	0
YPL085W	SAKL0H09570g	0	1	0
YPR169W	SAKL0F15730g	0	1	0
YLR014C	SAKL0G11902g	0	1	0
YLR335W	SAKL0B03850g	0	2	0
YHR156C	SAKL0C03036g	0	2	0
YDR169C	SAKL0H14630g	0	1	2
YJL047C	SAKL0D07458g	1	0	0
YIL121W	SAKL0E08778g	0	2	0
YJL071W	SAKL0D06336g	0	1	0
YNR015W	SAKL0A02068g	0	1	0
YHR099W	SAKL0G07458g	0	1	0
YHR063C	SAKL0G09438g	0	1	0
YNL103W	SAKL0E10428g	2	0	4
YNL311C	SAKL0C13002g	0	2	0
YNL256W	SAKL0C03278g	0	1	0
YBR015C	SAKL0C10472g	0	1	0
YER184C	SAKL0C00242g	0	2	0
YHR022C	SAKL0G11154g	0	2	0
YNL281W	SAKL0C09460g	0	1	0
YKL070W	SAKL0A00176g	0	1	0
YOL063C	SAKL0C07304g	0	3	0
YML029W	SAKL0G05962g	0	1	0
YGR172C	SAKL0G13552g	0	1	0
YOR274W	SAKL0H05368g	0	1	0
YJR113C	SAKL0F14982g	0	1	0
YLR127C	SAKL0H15598g	0	1	0
YER172C	SAKL0H17754g	0	1	4
YOR039W	SAKL0G01672g	0	1	0
YMR101C	SAKL0E03476g	0	1	0
YNL325C	SAKL0C13552g	0	1	0
YMR150C	SAKL0E05478g	0	1	0
YNL186W	SAKL0E13002g	0	1	0
YKL015W	SAKL0G19470g	0	2	0
YLR097C	SAKL0H16588g	0	1	0
YML037C	SAKL0G06226g	0	1	0
YCL056C	SAKL0C00572g	0	1	0

YGR276C	SAKL0H01034g	0	2	0
YHR184W	SAKL0H01826g	0	1	0
YHR086W	SAKL0G08118g	0	2	4
YJL081C	SAKL0D05874g	0	1	0
YKR045C	SAKL0C07084g	0	3	0
YPR140W	SAKL0F03212g	0	1	0
YML049C	SAKL0D12342g	0	3	0
YKR054C	SAKL0C06446g	0	2	0
YLR139C	SAKL0H15004g	0	1	0
YHR032W	SAKL0G10934g	0	0	3
YHR168W	SAKL0H02200g	0	1	0
YHR062C	SAKL0G09482g	0	1	0
YHR084W	SAKL0G08184g	0	1	0
YAL032C	SAKL0B10450g	0	1	0
YDL159W	SAKL0F11110g	1	0	3
YGR125W	SAKL0F02926g	0	1	0
YMR240C	SAKL0B05610g	0	1	0
YKL130C	SAKL0E03806g	0	1	0
YOR073W	SAKL0G00660g	0	0	1
YGR047C	SAKL0C02816g	0	1	0
YLR191W	SAKL0F11748g	0	1	0
YCR027C	SAKL0D10780g	0	1	0
YLR079W	SAKL0B00572g	0	1	0
YML054C	SAKL0G16852g	0	0	2
YIL046W	SAKL0F08404g	0	0	2
YLR059C	SAKL0B01232g	0	2	0
YAL001C	SAKL0H22550g	0	1	0
YNL042W	SAKL0E08074g	1	0	2
YOR202W	SAKL0H08228g	0	1	0
YDR192C	SAKL0H13662g	0	2	0
YFR032C	SAKL0F07326g	1	1	1
YOL107W	SAKL0C09020g	0	1	0
YKR064W	SAKL0C05918g	0	1	0
YDR495C	SAKL0C09306g	0	1	0
YNL054W	SAKL0E08426g	3	1	5
YGL131C	SAKL0A04092g	0	4	0
YKR063C	SAKL0C05940g	0	1	0
YPL083C	SAKL0H09636g	0	1	0
YJL089W	SAKL0D05654g	0	1	0
YKL116C	SAKL0E03344g	0	1	0
YPL014W	SAKL0B02618g	0	2	0
YOR022C	SAKL0G06776g	0	1	0
YGL064C	SAKL0A04224g	0	1	0
YKL073W	SAKL0B11990g	0	1	0
YPL042C	SAKL0H11352g	0	1	0
YMR211W	SAKL0H05390g	0	1	0
YBL037W	SAKL0B06952g	0	1	0
YJR152W	SAKL0G16456g	0	1	0
YML117W	SAKL0D01364g	0	3	0
YDL220C	SAKL0E13266g	0	2	0
YFR036W	SAKL0F07590g	0	0	1
YER093C	SAKL0F12144g	1	0	4
YDR464W	SAKL0G06314g	0	3	0

YJR004C	SAKL0G12320g	0	1	0
YJR092W	SAKL0F14124g	0	2	0
YKL096W	SAKL0E02706g	0	3	0
YGR072W	SAKL0C08338g	0	1	0
YLR213C	SAKL0F10626g	0	2	0
YLR054C	SAKL0B01694g	0	1	0
YLR436C	SAKL0G17138g	0	1	0
YOR354C	SAKL0B11132g	0	1	0
YOR242C	SAKL0H06710g	0	1	0
YOR132W	SAKL0G03366g	0	1	0
YDR349C	SAKL0G07524g	0	2	0
YKR031C	SAKL0D04972g	0	2	0
